# Bases anatomiques des lésions de l’artère pudendale externe lors de la chirurgie des varices du membre pelvien

**DOI:** 10.11604/pamj.2016.24.199.7874

**Published:** 2016-07-07

**Authors:** Magaye Gaye, Assane Ndiaye, Papa Adama Dieng, Aynina Ndiaye, Papa Salmane Ba, Souleymane Diatta, Amadou Gabriel Ciss, Jean Marc Ndiaga Ndoye, Mamadou Diop, Abdoulaye Ndiaye, Mouhamadou Ndiaye, Abdarahmane Dia

**Affiliations:** 1 Laboratoire d’Anatomie et Organogenèse, Faculté de Médecine, de Pharmacie et d’Odontologie, Université Cheikh AntaDiop de Dakar, Sénégal; 2 Service de Chirurgie Thoracique et Cardiovasculaire, CHNU de FANN, Sénégal; 3 Laboratoire d’Anatomie et d’Organogenèse, UFR de Santé-Université Assane SECK de Ziguinchor, Sénégal

**Keywords:** Artère pudendale externe, grande veine saphène, dysfonction sexuelle, External pudendal artery, great saphenous vein, sexual dysfunction

## Abstract

**Introduction:**

L’artère pudendale externe est une branche collatérale de l’artère fémorale commune qui est destinée à la vascularisation du pénis ou du clitoris. Ses rapports avec la crosse de la grande veine saphène et de ses afférences, dans le trigone fémoral, sont très étroits. Cette situation fait qu’elle est souvent lésée lors de la crossectomie et de l’éveinage de la grande veine saphène. Ces lésions peuvent être à l’origine d’une dysfonction sexuelle.

**Méthodes:**

Il s’agit d’une dissection de 22 régions inguinales chez 13 hommes et 9 femmes qui ont bénéficié d’un abord chirurgical du trigone fémoral. La distribution et les rapports de l’artère pudendale externe par rapport à la crosse de la grande veine saphène sont étudiés.

**Résultats:**

L’artère pudendale externe unique est la plus fréquente. Toutes les artères pudendales externes ont pour origine l’artère fémorale commune. Le rapport le plus fréquent est le sous croisement de la crosse de la grande veine saphène par une artère pudendale externe unique. Par ailleurs, on a un précroisement, un croisement alterné et des rapports avec la veine fémorale commune et des afférences de la crosse de la grande veine saphène. Certaines techniques chirurgicales exposent plus ou moins à une lésion de l’artère pudendale externe.

**Conclusion:**

Ce travail confirme les données antérieures mais montre encore quelques particularités sur les rapports entre la crosse de la grande veine saphène et l’artère pudendale externe.

## Abstract (en)

**Introduction:**

External pudendal artery is a collateral branch of the common femoral artery which is the primary blood supply to the penis or the clitoris. Its relationship with the venous arch of the great saphenous vein and its afferents in femoral triangle, are very narrow. This often entails injuries during crossectomy and great saphenous vein stripping. These lesions can cause sexual dysfunction.

**Methods:**

This study reports the case of a dissection of 22 inguinal regions in 13 men and 9 women who underwent surgical treatment of the femoral triangle. The distribution and the relationship between the external pudendal artery and the venous arch of the great saphenous vein are studied.

**Results:**

Single external pudendal artery is the most common. All external pudendal arteries originate from the common femoral artery. The most common type of relationship is between the sub-crossing of the venous arch of the great saphenous vein and a single external pudendal artery. Moreover, we recorded a pre-crossing, an alternate crossing and some relationship with the common femoral vein and the afferences of the venous arch of the great saphenous vein. Some surgical techniques expose more or less to external pudendal artery injury.

**Conclusion:**

This study confirms previous data but shows some peculiarities about the relationship between the venous arch of the great saphenous vein and external pudendal artery.

## Introduction

L'impuissance sexuelle ou dysfonction érectile consiste, soit dans l'impossibilité durable d'obtenir une érection valable, soit de ne pas pouvoir la maintenir si elle est obtenue avec une rigidité pénienne suffisante pour l'accomplissement de l'acte sexuel au moment précis de l'intromission [1]. Sa fréquence est difficile à déterminer en raison d'une sous-estimation probable des cas déclarés et la définition donnée à la dysfonction érectile. L'index international de la dysfonction érectile permet d'être objectif sur l'étude de la prévalence de cette dernière [1]. Ainsi, en France, plus de 30% des hommes de plus de 40 ans présentent une impuissance sexuelle [2]. Un même ordre de grandeur est retrouvé en Amérique [3]. Au Nigéria, sur une étude portant sur 450 patients d'âge compris entre 18 et 70 ans, on dénombre 55,1% présentant une dysfonction érectile. Et selon la sévérité, on a respectivement 32,6%; 17,8% et 4,7% de dysfonction érectile minime, modérée et sévère [4]. Cette prévalence augmente avec l'âge, le statut matrimonial, la classe sociale et la pratique de l'activité physique [1, 4] mais varie aussi avec l'existence ou non d'un facteur de risque comme le diabète sucré, l'hypertension artérielle, la maladie ulcéreuse, le tabagisme, l'insuffisance rénale chronique et l'antécédent de chirurgie prostatique [5, 6]. Sur le plan étiologique, on distingue les dysfonctions érectiles fonctionnelles souvent psychologiques, organiques avec des lésions anatomo-pathologiques et mixtes associant les deux derniers [7]. Parmi les causes organiques, les maladies cardio-vasculaires et le diabète sucré sont au premier plan [6]. Les mécanismes d'action de ces pathologies sont liés à une atteinte neurologique et à une insuffisance circulatoire artérielle. Cette dernière est soit secondaire à un hypodébit cardiaque associé ou non à une occlusion ou à une sténose des artères destinées aux organes génitaux. Ces atteintes sont souvent secondaires à l'athérosclérose. Par ailleurs, l'insuffisance circulatoire artérielle peut être congénitale par agénésie des artères à destinée génitale [6]. La vascularisation artérielle du pénis est assurée principalement par des branches de l'artère pudendale interne et accessoirement par celles de l'artère pudendale externe [8]. De par une cause acquise ou congénitale, cette vascularisation peut être assurée exclusivement par les branches de l'artère pudendale externe. L'atteinte dégénérative au cours d'une maladie chronique ou un traumatisme, par exemple chirurgical, d'une artère pudendale externe dominante peut entraîner une difficulté d'érection par diminution de l'afflux de sang dans les corps caverneux [9].

Sur le plan anatomique, l'artère pudendale externe présente des rapports variables mais très étroits avec la crosse de la grande veine saphène [10]. Cette dernière est disséquée et sectionnée dans le cadre de la chirurgie des varices du membre pelvien. Ainsi au moment de l'abord du trigone fémoral ou de la dissection de la crosse et du tronc de la grande veine saphène, l'artère pudendale externe peut être ligaturée avec les afférences de la crosse ou complètement sectionnée. En 1980, Reinharez [11] publiait un article intitulé « réflexions au sujet d'une impuissance sexuelle » dans lequel il incriminait, sans pouvoir le justifier, la cure chirurgicale des varices, dans cette pathologie. En 1984, deux cas d'impuissance sexuelle, après éveinage de la grande veine saphène, sont publiés. Par ailleurs, la prédominance, dans certains cas, des artères pudendales externes, dans la vascularisation des organes érectiles est rapportée, dans la littérature [12]. C'est entre 1984 et 1985 que la première étude, sur les rapports entre l'artère pudendale externe et la crosse de la grande veine saphène, a été réalisée par l'équipe précédente ayant publié les deux cas d'impuissance sexuelle après éveinage [10]. Depuis lors, aucune étude n'est venue confirmer ces découvertes. Ainsi, nous avons décidé de réaliser une étude descriptive plus exhaustive pour dénombrer le nombre d'artère pudendale externe, décrire les rapports entre l'artère pudendale externe et la crosse de la grande veine saphène et de dégager les conditions dans lesquelles cette artère risque d'être lésé.

## Méthodes

Nous avons disséqué 22 régions inguino-fémorales dont 11 à droite (06 femmes et 05 hommes) et 11 à gauche (06 hommes et 05 femmes) chez 22 sujets. Tous ces patients avaient bénéficié d'un abord du trigone fémoral dont les indications étaient: une crossectomie de la grande veine saphène associée à un stripping et des ligatures étagées pour varices du membre pelvien chez 12 patients; une cure conservatrice et hémodynamique de l'insuffisance veineuse en ambulatoire (CHIVA) chez un patient pour varices du membre pelvien; un pontage fémoro-poplité pour artériopathie oblitérante des membres inférieurs chez 6 patients; une endartériectomie fémorale pour une plaque athéromateuse sténosante chez 2 patients; une canulation fémorale dans le cadre d'une circulation extra-corporelle chez un patient.

Ces interventions chirurgicales étaient réalisées par six chirurgiens séniors, à la clinique de Chirurgie Thoracique et Cardiovasculaire du centre national hospitalier et universitaire de FANN. Les patients étaient installés en décubitus dorsal sous une rachi-anesthésie ou une anesthésie générale avec une intubation oro-trachéale. Les membres supérieurs étaient étendus sur un appui-bras avec une abduction à 90^°^. L'abord inguinal se faisait par une incision cutanée qui était verticale à l'union 1/3 interne et 1/3 moyen du pli inguinal quand il s'agissait d'un pontage fémoro-poplité, d'une endartériectomie ou d'une canulation fémorale. Par contre, l'incision était horizontale lorsqu'il s'agissait d'une chirurgie de varices. Celle-ci se faisait 1 cm au-dessous du pli inguinal. La longueur des incisions verticale et horizontale variait entre 4 et 6 cm. L'abord se continuait par une dissection et hémostase du tissu cellulaire sous cutané. La grande veine saphène était disséquée, à ce stade, et était libérée jusqu'à sa crosse avec dissection et isolement des afférences de la crosse. Pour les gestes sur les artères fémorales, l'aponévrose état incisée permettant de disséquer et d'isoler ces dernières. Tous les vaisseaux étaient mis sous lacs. l'artère pudendale externe était recherchée, disséquée et mise sous lac. Avant la réalisation du geste chirurgical, le chirurgien précisait, pour l'artère pudendale externe, le nombre, l'origine, son trajet et ses rapports avec la crosse de la grande veine saphène, des afférences de la crosse et des vaisseaux fémoraux. A la fin de l'intervention, il réalisait un schéma des vaisseaux fémoraux avec la crosse de la grande veine saphène, les afférences de cette dernière et l'artère pudendale externe dans ses rapports avec la crosse de la veine grande saphène. Sur ce schéma, il précisait le nom et prénom du malade, son âge, son sexe, la pathologie du malade, le type d'intervention réalisé et le côté opéré.

## Résultats

### L'artere pudendale externe

**Figure 1:**
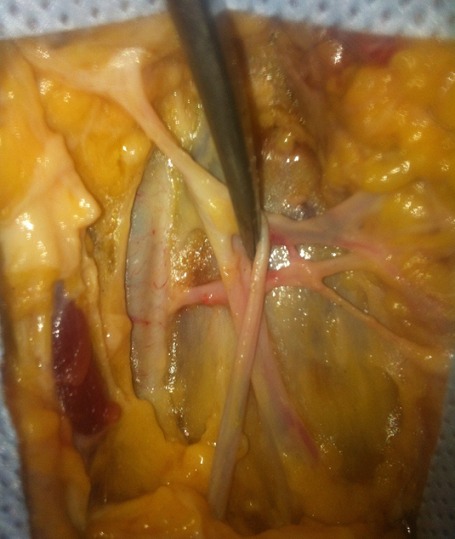
Naissance de l’artère pudendale externe sur l’artère fémorale commune

**L'origine**: Toutes les artères pudendales externes naissaient de l'artère fémorale commune (Figure 1).

**Le nombre** (Tableau 1, Tableau 2): chez 15 patients on n'avait qu'une seule artère pudendale externe. Selon le sexe, on avait 07 hommes et 08 femmes et pour les côtés, on découvrait une artère pudendale externe unique 9 fois à droite et 06 fois à gauche. Par ailleurs, avec le sexe associé au côté, on a chez les hommes une artère pudendale externe 6 fois à droite et 1 fois à gauche; chez les femmes on l'avait 3 fois à droite et 5 fois à gauche. Pour 06 patients, on avait 02 artères pudendales externes (artère pudendale externe supérieure et artère pudendale externe inférieure). Par rapport au sexe, on les voyait chez 05 hommes et chez 01 femme. On avait cette disposition 02 fois à droite et 04 fois à gauche. Avec le sexe combiné au côté, on la trouvait chez les hommes 4 fois à gauche et une fois à droite chez un homme et une femme. Chez une patiente on avait, à gauche 3, artères pudendales externes (supérieure, inférieure et moyenne ou accessoire).

**Tableau 1:** Nombre d’artères pudendales externes (APE) par rapport au sexe

Nombres d’APE	Homme	Femme
1	7	8
2	5	1
3	0	1

**Tableau 2:** Nombres d’artères pudendales (APE) externes par rapport au côté

Nombres d’APE	Droite	Gauche
1	9	6
2	2	4
3	0	1

**La distribution**: Chez 2 patients, on avait un tronc artériel commun naissant de l'artère fémorale commune qui donnait les deux artères pudendales externes supérieure et inférieure. Cette disposition était vue du côté gauche. Ils étaient tous les deux du sexe masculin. On constatait 2 artères pudendales supérieure et inférieure séparées avec chacune son origine sur l'artère fémorale commune chez 04 patients. Selon les côtés, on déterminait 02 à gauche et 02 à droite. Pour cette disposition, on avait 03 hommes et 01 femme. On avait, chez une patiente, un tronc artériel commun donnant trois artères pudendales (supérieure, inférieure et moyenne ou accessoire) à gauche. Chez 15 patients, on avait une artère pudendale externe unique avec un trajet direct vers les organes génitaux externes. Il s'agissait de 8 femmes et de 7 hommes.

**La terminaison**: Toutes les artères pudendales externes se rendaient vers les organes génitaux externes.

**Rapports de l'artère pudendale externe avec la crosse de la grande veine saphène.**

**Figure 2:**
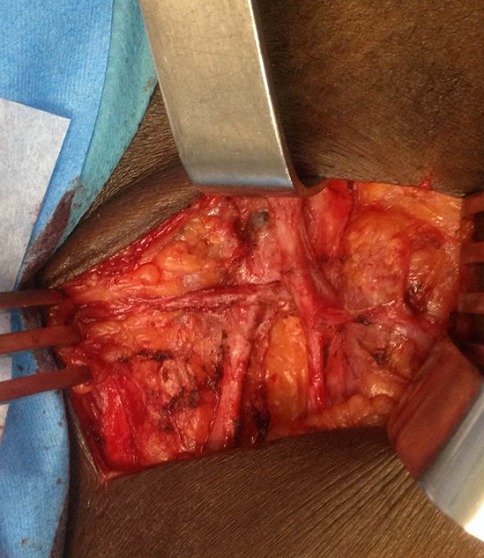
Pré-croisement de la grande veine saphène par l’artère pudendale externe

**Une artère pudendale externe unique qui pré-croise la crosse de la grande veine saphène sous les afférences de cette dernière (Figure 2).**

On retrouvait ce rapport dans 3 cas. On le notait 2 fois à droite et une fois à gauche. Deux de ces patients étaient des hommes dont l'un l'avait du côté gauche et l'autre du côté droit. La patiente l'avait du côté droit.

**Figure 3:**
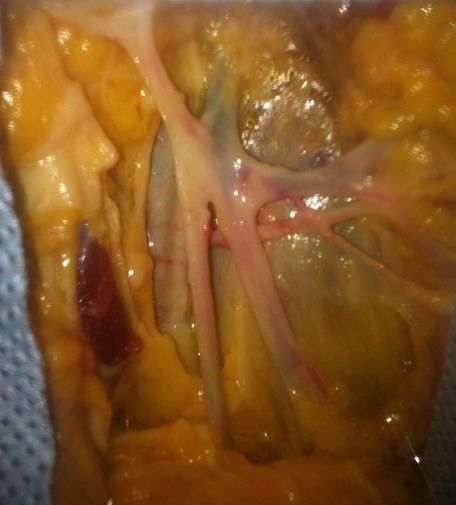
Sous-croisement de la grande veine saphène par l’artère pudendale

**Une artère pudendale externe unique qui sous-croise la crosse de la grande veine saphène sous les afférences de cette dernière (Figure 3).**

Cette disposition avait été retrouvée chez 08 patients. Cinq cas étaient du côté droit et 03 cas étaient du côté gauche. On avait, pour ce rapport, 4 patientes dont les 3 l'avaient du côté gauche et l'une du côté droit. Pour le sexe masculin, on avait 4 cas du côté droit.

**Un tronc artériel commun qui pré-croise le tronc de la grande veine saphène en dessous de la crosse avant de se bifurquer en deux artères pudendales externes supérieure et inférieure.**

Ce rapport était retrouvé chez un patient au côté gauche.

**Deux artères pudendales externes isolées (supérieure et inférieure) dont la supérieure et l'inférieure sous-croisent la crosse de la grande veine saphène.**

Ce rapport était retrouvé chez un patient au côté gauche.

**Deux artères pudendales externes isolées (supérieure et inférieure) dont l'inférieure sous-croise la crosse de la grande veine saphène et la supérieure passe au-dessus de la crosse entre les afférences de cette dernière et en avant de la veine fémorale commune.**

Cette disposition était retrouvée chez un patient à droite.

**Deux artères pudendales externes isolées (supérieure et inférieure) dont l'inférieure pré-croise la crosse de la grande veine saphène et la supérieure passe au-dessus de la crosse, entre les afférences de cette dernière et en avant de la veine fémorale commune.**

Ce rapport était chez deux patients de sexe opposé.

### Quelques situations anatomiques particulières

On avait retrouvé chez un patient, à droite, une grande veine saphène présentant un tronc unique avec une double crosse se jetant sur la veine fémorale commune et passage d'une artère pudendale externe unique dans cette boucle formée par les deux crosses et le tronc de lagrande veine saphène. On notait chez un homme, à gauche, un sous-croisement du tronc de la grande veine saphène par une artère pudendale externe unique au-dessus de l'abouchement dans la grande veine saphène accesoire de la veine pudendale externe. On constatait chez une femme, à droite, un pré-croisement de la veine fémorale commune par une artère pudendale externe unique au-dessus de la crosse de la grande veine saphène. Il y'avait chez une patiente, à gauche, un sous-croisement de la crosse de la grande veine saphène par une artère pudendale externe unique au-dessus des abouchements de la grande veine saphène accessoire et de la veine pudendale externe. On décrivait chez une patiente, à gauche, un tronc artériel pudendal commun qui naissait de l'artère fémorale commune et présentait une trifurcation avec un trajet ascendant des branches (artères pudendales externes supérieure, inférieure et moyenne ou accessoire) entre les veines afférentes de la crosse de lagrande veine saphène.

## Discussion

### L'artère pudendale externe

**Le nombre:** La présence d'une seule artère pudendale externe est la plus fréquente. Ainsi Henriet [10] montre que, parmi ces 256 patients ayant bénéficié d'un éveinage, 80% des femmes et 65% des hommes présentent une seule artère pudendale externe. Ces résultats confirment les nôtres avec la présence d'une seule artère pudendale externe dans la majorité des cas. La présence de deux artères pudendales externes habituellement notée, dans les ouvrages d'anatomie, occupe la deuxième place. Dans notre étude, nous la découvrons dans 6 cas sur 22, avec une prédominance masculine et du côté gauche. Henriet [10] retrouve aussi cette deuxième position avec une prédominance masculine, mais du côté droit. L'existence de trois artères pudendales est exceptionnellement rapportée dans la littérature. Nous avons retrouvé, dans notre série, un tronc artériel commun qui trifurque en trois branches artérielles que nous nommons artères pudendales externes supérieure, moyenne et inférieure. Cette même constatation est faite par Henriet [10] qui, en plus, a signalé une autre disposition rare à savoir trois artères pudendales externes naissant isolément de l'artère fémorale commune.

L'origine: Dans notre série, toutes les artères pudendales externes ont pour origine l'artère fémorale commune. LA FALCE [13] confirme cette origine exclusive sur l'artère fémorale commune, à partir d'une série de dissection de 50 régions inguino-fémorales sur des sujets anatomiques. Par contre, une origine au niveau de l'artère fémorale profonde est décrite par deux auteurs. Donnelly [14] montre, sur une série de 2080 régions inguinales disséquées, que 4,6% d'artères pudendales externes ont une origine sur l'artère fémorale profonde. Par ailleurs, Tanyeli [15] décrit cette même origine sur un cas isolé de dissection.

**La distribution:** La majorité de nos patients présente une artère pudendale externe unique. Cette prédominance est retrouvée, ailleurs, avec 77% chez les femmes et 63,7% chez les hommes [10]. Par contre, La Falce [13] retrouve une artère pudendale externe unique dans 30% de ses 46 régions inguino-fémorales disséquées. Selon le côté, nous notons une légère prédominance droite, chez les patients qui présentent une artère pudendale externe unique. Henriet [10] trouve une majorité à gauche avec 71,7% des patients dans cette situation. Le tronc artériel unique qui donne deux artères pudendales externes supérieure et inférieure n'est pas fréquent. La Falce [13] retrouve cette configuration chez 24% de ses dissections. La disposition particulière montrant un tronc artériel qui se divise en trois artères pudendales supérieure, moyenne et inférieure semble exceptionnel, dans la littérature, parce que n'ayant été décrite, une fois, que par un seul auteur [10]. L'existence de deux artères pudendales externes supérieure et inférieure qui ont des origines séparées sur l'artère fémorale commune est assez fréquente. Nous l'avons retrouvé chez deux patients. Henriet [10] l'a notée chez 15 patients sur ses 149 patients. Par contre La Falce [13] l'a remarquée un peu plus fréquemment, avec 21cas sur 46 artères pudendales externes retrouvés. La disposition exceptionnelle illustrée par trois artères pudendales externes supérieure, moyenne et inférieure qui ont des origines séparées sur l'artère fémorale commune a été décrite chez une patiente par un seul auteur [10].

### Rapports de l'artère pudendale externe avec la crosse de la grande veine saphène

Les rapports de l'artère pudendale externe et de la crosse de la grande veine saphène dépendent de l'anatomie de cette dernière. La disposition modale consiste en un tronc veineux saphénien qui se jette, dans la veine fémorale commune, par une crosse. Cette dernière reçoit des afférences que sont généralement les veines pudendales externes, la veine circonflexe iliaque superficielle et la veine épigastrique superficielle. La grande veine saphène peut être dédoublée avec un tronc veineux saphénien qui se termine par la crosse et une veine grande saphène accessoire qui se jette dans la première [14]. Par ailleurs, on peut voir un tronc veineux saphénien avec deux crosses qui se jettent dans la veine fémorale commune comme nous l'avons retrouvé. Ndiaye [16], sur une série de 54 régions inguinales disséquées, a retrouvé 4 doubles crosses de la grande veine saphène. Dans notre série, le rapport le plus fréquent est l'artère pudendale externe unique qui sous croise la crosse de la grande veine saphène. Cette fréquence est confirmée, par la seule étude exhaustive sur le rapport de l'artère pudendale externe, avec 70,4% des cas [10]. Par ailleurs, avec ce rapport, on ne note pas de prédominance de côté ni de sexe. Ndiaye [16] retrouve, dans sa série, 50% d'artères pudendales externes qui sous croisent la crosse de la grande veine saphène. Ce rapport expose à des lésions de l'artère pudendale externe, lors de la dissection de la partie proximale du tronc de la grande veine saphène. On peut aussi confondre, au moment de la ligature des afférences veineuses de la crosse de la grande veine saphène, la partie terminale de l'artère pudendale externe et la veine pudendale externe satellite. Le deuxième rapport le plus rencontré, dans notre étude, est l'artère pudendale externe unique qui précroise la crosse de la veine grande saphène. Henriet [10] retrouve le même rang avec un taux à 36,9%. Par ailleurs, dans une série de 1527 artères pudendales externes identifiées représentant 73,1% des dissections, 16,8% des artères précroisent la crosse de la grande veine saphène [14]. Ce pré-croisement rend l'artère pudendale externe proche du tissu cellulaire sous-cutané. De ce fait, elle peut être lésée lors de la dissection du tissu cellulaire sous-cutané pour accéder à la crosse de la veine grande saphène.

Les autres rapports dépendent de la morphologie de l'artère pudendale externe. Nous avons noté le tronc unique qui précroise la crosse de la veine grande saphène avant de donner les deux artères pudendales externes. On a noté aussi le tronc unique qui, avant d'atteindre la crosse, se divise et l'entoure comme une pince. Cette dernière disposition est décrite une fois par Ndiaye [16]. Les artères pudendales externes qui naissent isolément de l'artère fémorale commune et qui contractent, sur leurs trajets, des rapports différents et alternés, en termes de précroisement et de sous-croisement, sont aussi décrites par les auteurs [10, 16]. Par ailleurs, le cas de trois artères pudendales externes isolées qui précroisent la crosse de la veine grande saphène est décrit une seule fois [10]. Par contre, dans notre série, nous avons retrouvé un tronc artériel commun donnant trois artères pudendales supérieure, moyenne et inférieure passant entre les afférences de la crosse de la grande veine saphène. Cette situation expose aux blessures, lors des dissections et ligatures des afférences veineuses de la crosse. Les rapports inhabituels dus au trajet haut situé ou bas situé de l'artère pudendale externe, à la trifurcation de cette dernière et au dédoublement de la grande veine saphène. Ces variations changent logiquement les rapports avec la crosse et font surgir d'autres types de rapports comme avec la veine fémorale commune, les afférences de la crosse de la grande veine saphène et la veine saphène accessoire. Donnelly [14] décrit une artère pudendale externe qui passe entre une grande veine saphène et une veine saphène accessoire dans 4,6% de ses observations. Henriet [10] décrit, quand à lui, une artère pudendale externe qui passe entre la grande veine saphène et la veine de Jacomini. Ces situations accroissent la difficulté chirurgicale et en même temps le risque de blessure de l'artère pudendale externe.

## Conclusion

L'artère pudendale externe est une branche de l'artère fémorale commune. Sa situation et son trajet se trouvent essentiellement au niveau du trigone fémoral. Au niveau du trigone fémoral, l'artère pudendale externe contracte des rapports étroits avec la grande veine saphène. Ainsi lors de la chirurgie des varices du membre pelvien, l'artère pudendale externe peut être lésée, entrainant des fois une dysfonction érectile par insuffisance circulatoire des organes génitaux externes. Au vu de nos résultats, nous recommandons une étude plus exhaustive analytique sur cette artère et ses rapports avec la grande veine saphène, mais, aussi une étude anatomo-clinique et de prévalence du dysfonctionnement érectile après cure de varices. Sur le plan chirurgical, nous préconisons de rechercher jusqu'à trois artères pudendales externes tout en sachant que l'artère pudendale unique est la plus fréquente; de chercher cette artère aussi bien entre les afférences de la crosse qu'à la hauteur du tronc de la grande veine saphène. Cette attitude permettra d'éviter de léser l'artère pudendale externe, lors de la dissection et de la ligature du tronc et des afférences de la grande veine saphène.

### Etat des connaissances actuelles sur le sujet

- Les rapports de la crosse de la grande veine saphène et l'artère pudendale externe;
- Le lien entre la dysfonction érectile et la lésion de l'artère pudendale externe.

### Contribution de notre étude à la connaissance

- Mécanismes de lésions de l'artère pudendale externe au moment de la chirurgie des varices;
- L'existence d'un tronc commun donnant naissance à trois artères pudendales externes.
